# Fabrication of Polycaprolactone/Nano Hydroxyapatite (PCL/nHA) 3D Scaffold with Enhanced In Vitro Cell Response via Design for Additive Manufacturing (DfAM)

**DOI:** 10.3390/polym13091394

**Published:** 2021-04-25

**Authors:** Yong Sang Cho, So-Jung Gwak, Young-Sam Cho

**Affiliations:** 1 Medical IT Convergence Research Section, Daegu-Gyeongbuk Research Center, Electronics and Telecommunications Research Institute (ETRI), Techno Sunhwan-ro 10-gil, Dalseong-gun, Daegu 42994, Korea; yongsangcho@etri.re.kr; 2 Department of Chemical Engineering, College of Engineering, Wonkwang University, 460 Iksandae-ro, Iksan, Jeonbuk 54538, Korea; 3 Department of Mechanical Design Engineering, College of Engineering, Wonkwang University, 460 Iksandae-ro, Iksan, Jeonbuk 54538, Korea

**Keywords:** bone tissue engineering, design for additive manufacturing, kagome structure, scaffold

## Abstract

In this study, we investigated the dual-pore kagome-structure design of a 3D-printed scaffold with enhanced in vitro cell response and compared the mechanical properties with 3D-printed scaffolds with conventional or offset patterns. The compressive modulus of the 3D-printed scaffold with the proposed design was found to resemble that of the 3D-printed scaffold with a conventional pattern at similar pore sizes despite higher porosity. Furthermore, the compressive modulus of the proposed scaffold surpassed that of the 3D-printed scaffold with conventional and offset patterns at similar porosities owing to the structural characteristics of the kagome structure. Regarding the in vitro cell response, cell adhesion, cell growth, and ALP concentration of the proposed scaffold for 14 days was superior to those of the control group scaffolds. Consequently, we found that the mechanical properties and in vitro cell response of the 3D-printed scaffold could be improved by kagome and dual-pore structures through DfAM. Moreover, we revealed that the dual-pore structure is effective for the in vitro cell response compared to the structures possessing conventional and offset patterns.

## 1. Introduction

Large bone defects in which natural self-healing is impossible may be caused by traffic accidents, bone tumors, or aging in human life [1,2,3]. To regenerate large bone defects, intervention therapy using bone-graft implants is required. Bone tissue engineering has attracted attention as a method of intervention therapy because of the intrinsic merits of tissue engineering. In bone tissue engineering, scaffolds that can provide a dynamic environment for cell growth are regarded as key items, along with cells and growth factors [4,5,6]. A scaffold should be biocompatible and biodegradable, and provide suitable mechanical properties, pore sizes, and well-interconnected pores [7,8,9]. Many studies using 3D-printing techniques, including stereolithography apparatus (SLA) [10], selective laser sintering (SLS) [11], and melting extrusion (3D plotting) [12], have been proposed for fabricating a scaffold satisfying the abovementioned requirements. The 3D-printing technique is known to enable customized 3D scaffolds with suitable pore size, porosity, and interconnected pore networks using biocompatible and biodegradable materials. Representative 3D-printable synthetic polymers with biocompatible and biodegradable properties are poly (ε-caprolactone) (PCL), poly (lactic acid) (PLA), poly (lactic-co-glycolide) (PLGA), and poly (glycolic acid) (PGA). The aforementioned synthetic polymers usually have low biological responses such as osteoconduction, compared to hydroxyapatite (HA), tricalcium phosphate (TCP), and bioactive glass. To overcome the limitations of synthetic polymers, 3D-printed composite scaffolds consisting of synthetic polymers and bioceramics have been proposed as bone grafts [13,14].

The dual-pore scaffold, with global and local pores, was proposed to enhance the in vitro cell response of 3D-printed scaffolds. They were fabricated by robocasting [15], microstereolithography (MSTL) [16], direct polymer melt deposition/electrospinning [17], and powder extruder systems [18]. Global pores can assist in providing oxygen and nutrients to living cells, and local pores are known to be an aid for cell adhesion [19]. However, the mechanical properties of the dual-pore scaffold are remarkably lower than those of the 3D-printed scaffold with the conventional pattern owing to higher porosity. Moreover, studies on the enhancement of the in vitro cell response of 3D-printed scaffolds via layer-down (0-60-120°, 0-45-90-135° and 0-30-60-90-120-150°) or offset patterns have been reported [20,21,22,23,24,25,26]. Although the cell adhesion and proliferation of the 3D-printed scaffold were improved, because of the increasing cell-seeding efficiency of the 3D-printed scaffold with layer-down or offset patterns, their mechanical properties were lower than those of the 3D-printed scaffold with a conventional pattern at similar porosity. Meanwhile, in our previous studies, a 3D-printed scaffold with a 3D open-cellular kagome structure and composite material consisting of polycaprolactone (PCL)/nano-hydroxyapatite (nHA) was proposed to enhance the mechanical properties of 3D-printed scaffolds [27]. The mechanical properties of the 3D-printed scaffold with a kagome structure were superior to those of the reported 3D-printed scaffold with a conventional pattern at a similar porosity. Furthermore, the in vitro cell response of the 3D-printed scaffold with a kagome structure improved compared with the 3D-printed scaffold with the conventional pattern at the same pore size and porosity. This is because the cell-seeding efficiency of the 3D-printed scaffold with a kagome structure was increased by the complex structure compared to the 3D-printed scaffold with the conventional pattern [28].

Therefore, a 3D-printed dual-pore kagome-structure PCL/nHA scaffold is proposed in this study to overcome the disadvantages of dual-pore scaffolds or 3D-printed scaffolds with layer-down patterns and offset patterns. The proposed structure can enhance the in vitro cell response with acceptable mechanical properties compared to the conventional 3D-printed scaffolds. The mechanical properties and in vitro cell response of the proposed 3D-printed dual-pore kagome-structure PCL/nHA scaffold were compared with those of 3D-printed PCL/nHA scaffolds with conventional and offset patterns at the same pore size and porosity. For simplicity, we have defined the nomenclature as “Conv 1 (similar pore size)”, “Conv 2 (similar porosity)”, “Offset 1 (similar pore size)”, “Offset 2 (similar porosity)”, and “dual-pore”. Additionally, dual-pore scaffolds reported in the literature were fabricated through the combination of the 3D-printing technique and conventional fabrication method (salt-leaching, solvent casting, non-solvent induced phase separation, etc.) [15,16,17,18]. However, the abovementioned methods are difficult to control pore size, pore shape, and porosity because local pores were formed by salt-leaching, solvent casting, non-solvent induced phase separation, etc. Therefore, we proposed a 3D-printed PCL/nHA scaffold with a dual-pore kagome structure because the 3D-printing technique can control the scaffold’s pore size, pore shape, and porosity. Moreover, to assess the influence of dual-pore structure fabricated by 3D-printing technique on mechanical property and in vitro cell response, the reported dual-pore scaffolds were excluded from a control group.

## 2. Materials and Methods

### 2.1. Design of PCL/nHA Scaffold with Dual-Pore Kagome Structure

**Figure 1:**
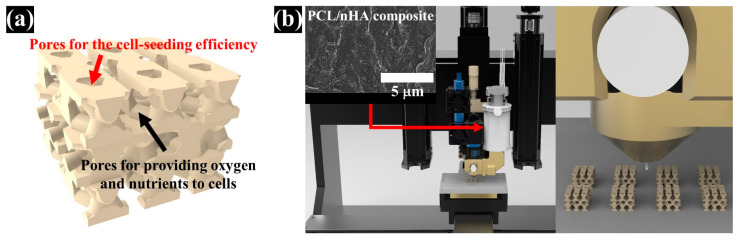
Schematics of the 3D-printed scaffolds with dual-pore kagome-structures: (**a**) 3D image of designed dual-pore scaffold; (**b**) 3D-printing system (material-extrusion system).

A PCL/nHA scaffold with a dual-pore kagome structure was designed using the CATIA program (Dassault systems, Vilacoublay, France). The characteristics of this structure are explained in Supplementary Figure S1. To investigate the design of the PCL/nHA scaffold with a dual-pore kagome-structure, the numerical compressive modulus of the 3D scaffold with various strand sizes was analyzed via a ABAQUS program (Dassault Systems, Providence, RI, USA) (Supplementary Figures S2 and S4). Additionally, the numerical compressive moduli of the control group scaffolds (Conv 1, Conv 2, Offset 1, and Offset 2) were calculated. The 3D finite element method (FEM) models consisted of quadratic tetrahedral elements (element type C3D10) in the ABAQUS program. The total number of nodes for Conv 1, Conv 2, Offset 1, Offset 2, and dual pore were 427,791, 357,452, 444,582, 361,868, and 590,640, respectively. The boundary conditions are described in Supplementary Figure S2. The Young’s modulus and Poisson’s ratio for the composite material (PCL/nHA 10 wt%) were assumed to be 398.7 MPa and 0.38, respectively, using the average value of the tensile modulus of the bulk composite material, with the same weight ratio for the fabricated scaffold (Supplementary Figure S3) [28]. The 3D dual-pore kagome-structure scaffold, possessing a numerical compressive modulus similar to that of a 3D scaffold with a conventional pattern at the same pore size, is defined by the following parameters: dimensions = 5 × 5 × 3.6 mm^3^, porosity = 60%, pore size = 500 μm, and strand size = 1.4 mm (Figure 1a and Table 1). The designed parameters of the control scaffolds with the same dimensions are listed in Table 1. The 3D-printing pathways were generated from the STL files of the designed 3D scaffold using an open-source STL-generating program (Slic3r, Rome, Italy).

### 2.2. Preparation and Fabrication of the PCL/nHA Scaffold with The Dual-Pore Kagome Structure

Commercially available nano-sized hydroxyapatite (Sigma-Aldrich, St. Louis, MO, USA) and polycaprolactone (Polysciences, Warrington, PA, USA) were used as 3D-printable materials. Our previous study describes the preparation of composite materials for 3D-printing systems in detail [27]. In brief, PCL pellets were dissolved in 5 *w*/*v*% dichloromethane (Daejung Chemicals and Materials, Siheung, Korea), followed by the addition of 10 wt% nHA powder to the 5 *w*/*v*% PCL solution. The solution was sonicated with a sonicator (Korotec, Seoul, Korea) at 450 W for 45 min to uniformly disperse the nHA particles in the PCL matrix. The dispersed solution was dried in a vacuum oven at 80 °C to remove the residual dichloromethane. The prepared composite material was melted in a stainless-steel barrel with a ceramic nozzle with an inner diameter of 100 μm at 100 °C for 1 h. The melted material was extruded by a single screw at 45 rpm, and the 3D scaffold with a dual-pore kagome structure was fabricated by a pathway created from the STL file simultaneously (Figure 1). The control scaffolds were fabricated using a nozzle with an inner diameter of 500 μm using the abovementioned method.

### 2.3. Characterization of the Fabricated Scaffolds

The chemical components of the fabricated composite material for the 3D-printing system were analyzed using Fourier transform infrared spectroscopy (JASCO, Tokyo, Japan). Two samples were investigated for each material type. To determine the actual amount of nHA particles in the fabricated scaffold, the weight ratio of the residual material at 600 °C was measured using a thermal gravimetric analyzer (TA Instruments, New Castle, DE, USA). For each scaffold type, the average value for three scaffolds was calculated. In the visualized graph, a value similar to the average value of the TGA data was selected. The morphologies of the fabricated scaffolds were observed at the top and side views using a field emission scanning electron microscope (Hitachi, Tokyo, Japan) to compare the morphological characteristics of the fabricated scaffolds. Two scaffolds were observed for each scaffold type. The apparent pore sizes and porosities of the fabricated scaffolds were compared using an optical microscope (Magic i, Seoul, Korea) and Equation (1) to investigate the structural characteristics of the fabricated scaffolds. Ten samples were measured for each type of scaffold.

(1) $$\;\mathrm{Porosity}\left(\%\right)=\frac{V_{0}-\left(\frac{wt_{\mathrm{PCL}}\times m}{\rho \mathrm{PCL}}+\frac{wt_{\mathrm{HA}}\times m}{\rho \mathrm{HA}}\right)}{V_{0}}\times 100$$

where *V*_0_ is the apparent volume of the fabricated scaffolds; m is the mass of the fabricated scaffold; *ρ*PCL and *ρ*HA are the densities of each material; *wt*_PCL_ and *wt*_HA_ are the weight ratios of PCL and HA, respectively, in the prepared composite material.

To compare the compressive moduli of the fabricated scaffolds, measurements were performed using a uniaxial testing machine (MTS, Eden Prairie, MN, USA) at a constant strain rate of 1 mm/min with a 5 kN loading cell. The compressive modulus was defined within 1% strain of the stress-strain curve via offset method because the scaffold does not show a well-defined yield point in compression. Ten samples were used for each type of scaffold.

### 2.4. Osteoblast-Like Cell Culture and Cell-Growth Analysis of the Fabricated Scaffolds

Human osteogenic sarcoma cells, Saos-2, (Korea Cell Line Bank, Seoul, Korea) were cultured in Dulbecco’s modified Eagle’s medium (Gibco, Grand Island, NY, USA) supplemented with 10% fetal bovine serum (Gibco, Grand Island, NY, USA), 2 mM L-glutamine, 100 U/mL penicillin, and 100 μg/mL streptomycin (Gibco, Carlsbad, CA, USA), and maintained in a humidified incubator at 37 °C with 5% CO_2_. The medium was changed every other day. To measure cell proliferation in the scaffold, cells were seeded into each scaffold at a density of 1 × 10^5^ cells/scaffold. Cell proliferation was measured using a cell counting kit (Invitrogen, Carlsbad, CA, USA). The absorbance of viable cells was measured at 450 nm using a microplate reader. The CCK-8 assay data were presented as optical density values from four different scaffolds.

The alkaline phosphatase (ALP) assay of cells in the scaffold was performed using the TRACP&ALP assay kit (TakaRa-Bio, Kusatsu, Japan). The cells (1 × 10^5^ cells/scaffold) were seeded into the scaffold and cultured for 14 days. The samples were washed with 0.9% NaCl in water and incubated with extraction buffer. The lysates of the samples were mixed with ALP substrate solution containing p-nitrophenyl phosphate (pNPP). After 1 h, the reaction was stopped by adding 50 μL of 1 M NaOH. The ALP activity was measured spectrophotometrically at 405 nm using a microplate reader. The total protein concentration was measured using a BCA assay kit (iNtRon Biotechnology, Seongnam, Korea). Four scaffolds were used in each case.

### 2.5. Statistical Analysis

The obtained data are indicated as the mean ± standard deviation. Statistical analysis of obtained data was performed using the Student’s t-test (Microsoft, Redmond, WA, USA). Differences were considered statistically significant at *p* < 0.05.

## 3. Results

### 3.1. Comparison of the Chemical Component and nHA Content in the Fabricated Scaffold

**Figure 2:**
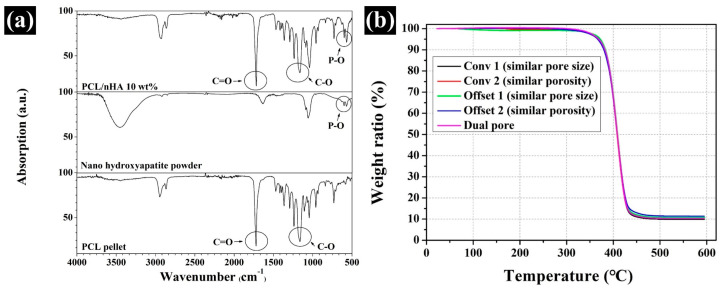
Comparison of the chemical composition and weight ratio: (**a**) FT-IR results for pure and PCL/nHA composite materials; (**b**) TGA results for the fabricated scaffolds.

The chemical compositions of the composite material and pure material were investigated via FT-IR (Figure 2a) to compare the chemical components of the prepared composite materials with each pure material. The C = O (1721) and C–O (1163) groups appeared in pure PCL. For pure nHA, the P-O (590) group was detected. In the case of the composite material, the C = O, C–O, and P-O groups appeared at the same wavenumber. In addition, the actual amount of nHA particles in the fabricated scaffolds from 30 °C to 600 °C were measured (Figure 2b). The measured weight ratios of Conv 1, Conv 2, Offset 1, Offset 2, and dual pore were 9.9 ± 0.5%, 10.6 ± 0.2%, 11.0 ± 0.4%, 10.9 ± 0.3%, and 10.3 ± 0.2%, respectively. The designed weight ratio of nHA particles in the composite material was similar to the actual content of nHA particles in the fabricated scaffolds (Figure 2b).

### 3.2. Comparison of the Morphological and Structural Characteristics of the Fabricated Scaffold

**Figure 3:**
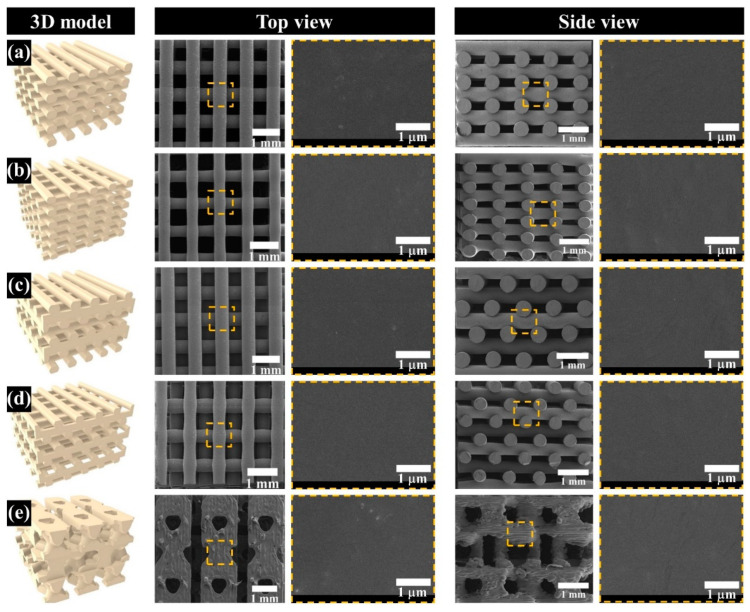
Three-D modeling, top-view, and side-view images of the fabricated scaffolds: (**a**) Conv 1 (similar pore size); (**b**) Conv 2 (similar porosity); (**c**) Offset 1 (similar pore size); (**d**) Offset 2 (similar porosity); (**e**) dual pore.

**Figure 4:**
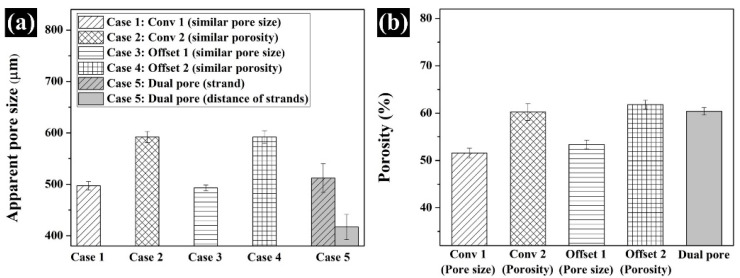
Structural characteristics of fabricated scaffolds: (**a**) Apparent pore size; (**b**) porosity.

The morphology of the proposed scaffolds was compared by observing the top- and side-view morphologies (Figure 3a–e). Interconnected pores and uniform pore sizes, similar to the designed 3D model in all scaffolds, were observed. For the Conv 1, Conv 2, Offset 1, and Offset 2 scaffolds, pores formed by the distance between strands were confirmed (Figure 3a–d). In contrast, in the case of the dual-pore scaffold, pores could be observed in the strand of the scaffold and by the distance between strands (Figure 3e). The apparent pore size and porosity were measured to analyze the structural characteristics of the proposed scaffolds (Figure 4). The apparent pore size and porosities of Conv 1, Conv 2, Offset 1, and Offset 2 were 497 ± 8 μm/51.5 ± 1.0%, 591 ± 10 μm/60.2 ± 1.7%, 493 ± 5 μm/53.3 ± 0.9%, and 591 ± 12 μm/61.7 ± 0.9%, respectively. For the dual-pore scaffold, the apparent pore size and porosity were measured as 512 ± 27 μm (pore size in the strand)/417 ± 24 μm (pore size by the distance of strands)/60.3 ± 0.8%. The measured apparent pore sizes and porosities of the fabricated scaffolds were consistent with those of the design parameters (Figure 4 and Table 1).

### 3.3. Assessment of the Compressive Modulus by Numerical and Experimental Analyses

**Figure 5:**
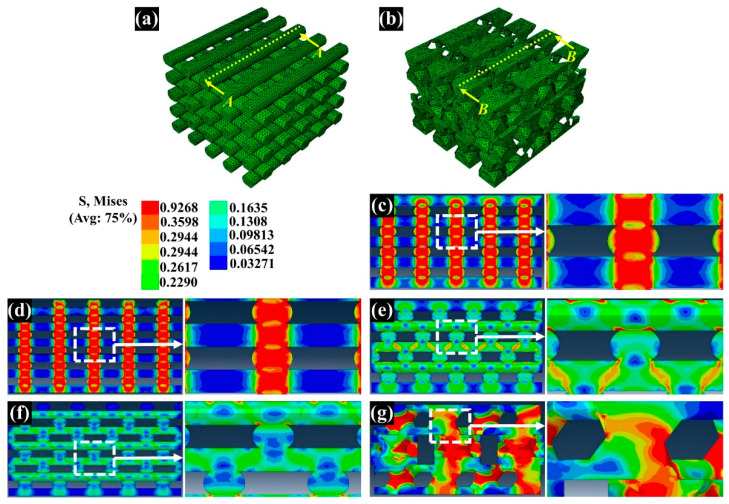
Deformed configuration and von Mises stress of the fabricated scaffold: (**a**) and (**b**) are the cross-sectional position of the designed scaffolds for the stress plot; (**c**–**g**) are the deformed configuration and von Mises stress of the designed scaffolds.

**Figure 6:**
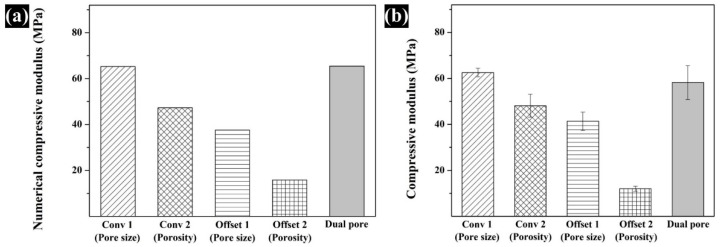
Comparison of the (**a**) numerical and (**b**) experimental compressive modulus for the fabricated scaffolds.

The compressive modulus of the fabricated scaffolds was assessed by a compressive test of the fabricated scaffolds performed via numerical and experimental analyses (Figure 5 and Figure 6). For the numerical analysis, the compressive modulus of Conv 1, Conv 2, Offset 1, Offset 2, and dual-pore scaffolds was calculated as 65.2 MPa, 47.2 MPa, 37.6 MPa, 15.8 MPa, and 65.4 MPa, respectively (Figure 6a). The compressive modulus of Conv 1, Conv 2, Offset 1, Offset 2, and dual-pore scaffolds in the experimental analysis via UTM were measured as 62.5 ± 1.8 MPa, 48.1 ± 4.9 MPa, 41.3 ± 3.9 MPa, 12.0 ± 1.1 MPa, and 58.2 ± 7.3 MPa (Figure 6b). The trends in the actual compressive modulus of the fabricated scaffolds were similar to the trends of the predicted value in the numerical compressive modulus. Furthermore, no significant differences between the actual compressive modulus of Conv 1 and the dual-pore scaffolds were observed.

### 3.4. Assessment of the In Vitro Cell Response on the Fabricated Scaffolds

**Figure 7:**
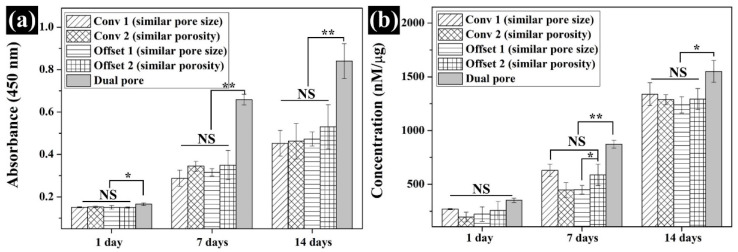
Comparison of the in vitro cell response of the scaffolds: (**a**) CCK-8; (**b**) ALP (NS: nonsignificant, * *p* < 0.05, ** *p* < 0.01).

To investigate the influence of dual-pore structure on the in vitro cell response, CCK-8 and ALP assays were performed for 14 days. On day 1, the values of cell attachment to the fabricated scaffolds were not different except for the dual-pore scaffold. The cell-adhesion ability of the dual-pore scaffold was slightly higher than that of the other scaffolds (Figure 7a). Moreover, 7 and 14 days after cell culture on the fabricated scaffolds, the cell growth of the dual-pore scaffold was dramatically increased compared to other scaffolds and was 1.5 times those of the control scaffolds. For the ALP assay, the concentrations of the fabricated scaffolds gradually increased over 14 days (Figure 7b). In the dual-pore scaffold, the concentration values rapidly improved at 7 and 14 days, similar to the results of the CCK-8 assay.

## 4. Discussion

In bone tissue engineering, bone tissue cells are known to prefer pore sizes from 300–500 μm. Since a pore size >300 μm is recommended to improve bone regeneration [29,30,31], we designed a 3D-printed scaffold with a pore size of 500 µm. In our previous study [27], the mechanical properties and in vitro cell response of the 3D-printed PCL/nHA scaffold with a kagome structure were assessed according to various nHA contents (3, 5, and 10 wt%). The abilities of the 3D-printed scaffold with nHA 10 wt% were superior to those of the other scaffolds (3 and 5 wt%). Moreover, the weight ratio (nHA 10 wt%) enabled the successful fabrication of a scaffold without 3D-printing problems, such as nozzle clogging or the nonuniform extrusion of composite materials. Hence, the weight ratio of the nHA particles in the composite material was fixed at 10 wt% in this study.

The chemical characteristics of the prepared composite material as 3D-printing material were not significantly different from those of the pure materials (PCL pellets and nHA particles) (Figure 2a). Moreover, the actual amount of nHA particles in all scaffolds was similar to the designed weight ratio of nHA particles in the composite material (Figure 2b). Therefore, the influence of the composite material on the compressive modulus and in vitro cell response can be excluded. The scaffolds fabricated by the 3D-printing system had uniform pore sizes and pore shapes, similar to those of the 2D and 3D CAD models (Figure 3). There was no difference in the morphological characteristics of the fabricated scaffolds, as shown in the high-magnification FE-SEM images (Figure 3). The apparent pore sizes and porosities of all the scaffolds were similar to those of the designed parameters, such as pore sizes of 500 μm and 600 μm/porosity of 50% and 60%, respectively (Figure 4). In the case of the dual-pore scaffold, the pore sizes in the strand were slightly lower than the designed parameter because of the 3D-printing deposition pathway for the prevention of undesired pores in the kagome structure [27]. In terms of mechanical properties, the compressive moduli of Conv 1, Conv 2, Offset 1, and Offset 2 scaffolds decreased with increasing porosity of the scaffolds (Figure 5 and Figure 6). Furthermore, the compressive moduli of the Offset 1 and Offset 2 scaffolds were lower than those of the Conv 1 and Conv 2 scaffolds at the same pore size and porosity. This phenomenon could be explained by the bending behavior in the Offset 1 and Offset 2 scaffolds. In the von Mises contour plots of Conv 1 and Conv 2 scaffolds (Figure 5c,d), the stress is concentrated in the stacked region at the same position as the strands, implying that the stacked region acts like a vertical column that bears almost compressive force. However, the von Mises contour plots of the Offset 1 and Offset 2 scaffolds (Figure 5e,f) show that the trend of von Mises stress is similar to that of bending rods. In general, the apparent rigidity (apparent modulus) of the vertical column is higher than that of the horizontal rod when the compressive force is directed downwards. In contrast, the mechanical properties of the dual-pore scaffold were similar to those of the Conv 1 scaffold, despite the relatively higher porosity. This result could be explained by the fact that the dual-pore scaffold effectively disperses stress by the kagome structure compared to other scaffolds (Figure 5g). For the in vitro cell response, the cell-adhesion ability of the dual-pore scaffold was slightly better than that of the other scaffolds at 1 day (Figure 7a). The cell growth of the dual-pore scaffold was superior to that of the control group scaffolds at 7 and 14 days. Although the cell-growth value of the Offset 2 scaffold was slightly higher compared to the other control scaffolds, the values did not differ significantly over 2 weeks. For the ALP assay, the value of the concentration for the dual-pore scaffold was enhanced compared to other scaffolds at 7 and 14 days, similar to the results of the CCK-8 assay (Figure 7b). Furthermore, the concentrations of Conv 1 and Offset 2 scaffolds were superior to those of the Conv 2 and Offset 1 scaffolds at 7 days owing to the preferable pore size or relatively higher pore interconnectivity. However, 14 days after cell culture on the control group scaffolds, there was no difference in ALP concentration.

According to the reported studies on 3D-printed scaffolds with various patterns, Ferreira et al. observed that the compressive modulus of 3D-printed PCL scaffolds with conventional patterns (52.1 MPa) was superior to the layer-down patterns of 0/60/120° (44.0 MPa) and 0/45/90/135° (16.2 MPa) at similar porosities [21]. Rabionet et al. revealed that the adhesion of MCF-7 breast carcinoma cells on a 3D-printed scaffold with layer-down patterns was enhanced when compared with a 3D-printed scaffold with a conventional pattern [24]. Park et al. and Yilgor et al. found that despite a reduction in the compressive modulus of a 3D-printed scaffold with an offset pattern, its in vitro cell response improved compared with the conventional pattern because of the increasing cell-seeding efficiency [25,26]. However, in this study, the cell-seeding efficiency and cell proliferation of the control group scaffolds did not differ according to 3D-printing patterns, including conventional and offset patterns (Figure 7a). Furthermore, the ALP concentrations of the control group scaffolds were similar at 14 days (Figure 7b). In contrast, the dual-pore scaffold was effective for cell adhesion, proliferation, and ALP activity compared to the scaffolds with conventional and offset patterns (Figure 7). Furthermore, the compressive modulus of the dual-pore scaffold was similar to that of the Conv 1 scaffold, despite the higher porosity (Figure 6). Additionally, the compressive modulus of cancellous bone (spongy bone) was reported as 50–150 MPa at 50–80% porosity [32,33,34]. The compressive modulus and porosity of the proposed scaffold with dual-pore kagome structure were similar to those of natural cancellous bone. Therefore, we conclude that the mechanical properties and in vitro cell response of 3D-printed scaffolds can be enhanced via kagome and dual-pore structures through DfAM. Moreover, we expect the cancellous bone with a freeform shape can be regenerated via the proposed scaffold technique. Compared to previous investigations focused on dual-pore scaffolds [15,16,17,18], suitable mechanical property, porosity, and enhanced *in-vitro* cell response can be achieved through the proposed 3D-printing technique without using an organic solvent and complex processes.

## 5. Conclusions

In this study, a 3D-printed dual-pore kagome-structure scaffold was proposed to improve the in vitro cell response and mechanical properties. The characteristics, mechanical properties, and in vitro cell response of the dual-pore scaffold were compared with those of Conv 1 (similar pore size), Conv 2 (similar porosity), Offset 1 (similar pore size), and Offset 2 (similar porosity) scaffolds. The dual-pore scaffold’s mechanical properties were superior to those of the Conv 2 and Offset 2 scaffolds at similar porosities. In the case of similar pore sizes, the compressive modulus of the dual-pore scaffold was similar to that of the Conv 1 scaffold despite the higher porosity because of the characteristics of the kagome structure. For the in vitro cell response, cell adhesion, cell growth, and ALP concentration of the dual-pore scaffold were superior to those of the control group scaffolds. Consequently, we found that the mechanical properties and in vitro cell response of the 3D-printed scaffold could be improved by kagome and dual-pore structures through DfAM. Moreover, we revealed that the dual-pore structure is effective for the in vitro cell response compared to the structures possessing conventional and offset patterns.

## Figures and Tables

**Table 1:** Design parameters of the fabricated PCL/nHA scaffolds.

	2D Image	3D Image	Target Pore Size	Target Porosity
Conv 1 (similar pore size)	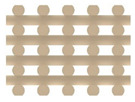	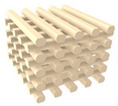	500 μm (distance of strands)	50%
Conv 2 (similar porosity)	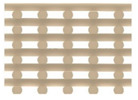	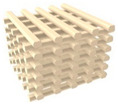	600 μm (distance of strands)	60%
Offset 1 (similar pore size)	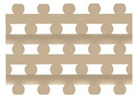	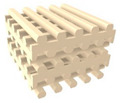	500 μm (distance of strands)	50%
Offset 2 (similar porosity)	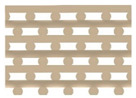	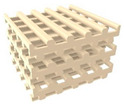	600 μm (distance of strands)	60%
Dual pore	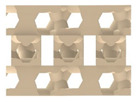	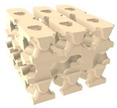	500 μm (strand)	60%
			500 μm (distance of strands)	

## Supplementary Materials

The following are available online at https://www.mdpi.com/article/10.3390/polym13091394/s1, Figure S1: Characteristics of the designed scaffold with dual-pore kagome-structure: (a) column region of the designed scaffold; (b) frame region of the designed scaffold; (c) top and side views of the designed scaffold, Figure S2: Boundary condition of numerical analysis: (a) control group scaffolds; (b) dual-pore scaffold, Figure S3: Tensile moduli of bulk PCL and composite bulk (PCL/nHA 10 wt%), and Figure S4: Numerical compressive moduli of dual-pore scaffolds with various strand sizes.
