# Supplementary material for: Fabrication of Polycaprolactone/Nano Hydroxyapatite (PCL/nHA) 3D Scaffold with Enhanced In Vitro Cell Response via Design for Additive Manufacturing (DfAM)
Source: Polymers (Basel). 2021 Apr 25;13(9):1394. doi: 10.3390/polym13091394 (PMC8123325; doi:10.3390/polym13091394)
Supplement: Supplementary file 1 [file polymers-13-01394-s001.zip › polymers-1178480-supplementary.pdf]

polymers

MDPI

Supplementary

Chapter 1. Characteristics of a scaffold with a dual-pore kagome structure and numerical analysis of the designed 3D scaffolds via ABAQUS

A scaffold with a dual-pore kagome structure was designed using the CAD program (CATIA version 5, Dassault Systems, Vilacoublay, France). The scaffold model with a dual-pore kagome-structure can be separated into columns and frames, as shown in Figures S1. Moreover, the scaffold consisted of pores with a kagome structure for cell-seeding efficiency and pore by channel to provide oxygen and nutrients to cells.

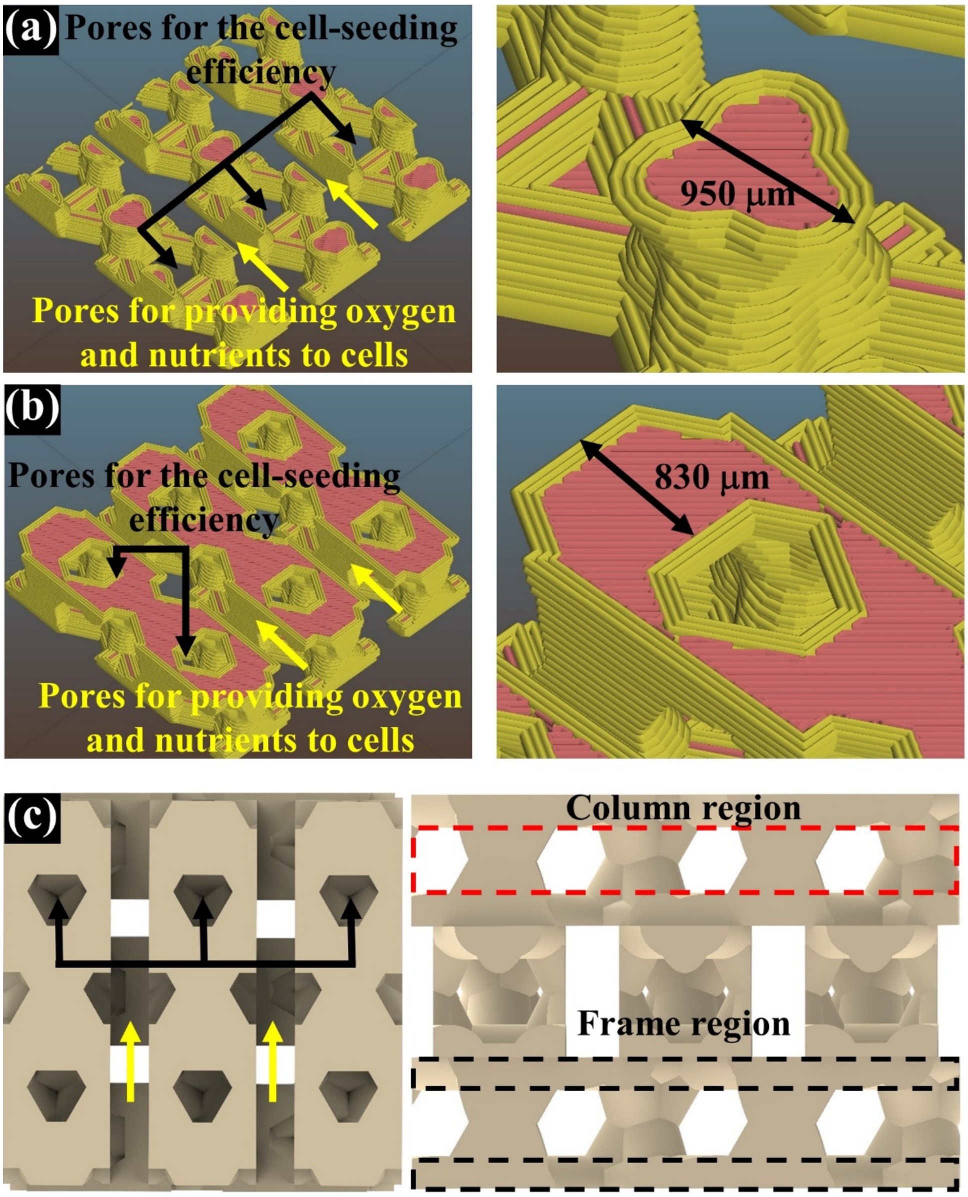

Figure S1. Characteristics of the designed scaffold with the dual-pore kagome-structure: (a) column region of the designed scaffold; (b) frame region of the designed scaffold; (c) top and side views of the designed scaffold.

To predict the compressive modulus of the designed 3D scaffolds, adequate boundary conditions for Conv 1, Conv 2, Offset 1, Offset 2, and dual-pore scaffolds were conducted, as shown in Figure S2. For the bottom surface area of the designed 3D scaffolds, every displacement of a single node in the x, y, and z directions was fixed as zero (indicated by a triangle). The line parallel to the x or y directions within

the indicated triangle is fixed as zero in the y/z direction or x/z direction, respectively (marked by a circle). The z direction of the other nodes on the bottom area was fixed at zero. To compress the designed 3D scaffolds, displacement in the z-direction is prescribed as -0.0036 mm at every node on the top area, i.e., 0.1% of the z-direction length of the designed 3D scaffold. The displacement boundary condition at the bottom surface area is a representative boundary condition for simple compression. The displacement conditions at the top surface area were selected to maintain the deformation of the scaffolds in the elastic region. Furthermore, in this numerical analysis, to implement the composite material properties (PCL/nHA 10 wt%), such as Young's modulus, the tensile modulus of the bulk composite (PCL/nHA 10 wt%) was measured, as shown in Figure S3. To measure the tensile modulus of the bulk composite, a dumbbell-shaped bulk composite with the following dimensions: overall length, 60 mm; width of grip section, 24 mm; gauge length, 22 mm; width, 2 mm; height, 1.2 mm was fabricated according to the ISO standard (KS M ISO 527-4: 2002). The tensile test was performed at a constant strain rate of 1 mm/min with a 5 kN loading cell using a universal testing machine (UTM; Model E42, MTS, Eden Prairie, MN, USA). The tensile modulus was determined from the initial linear region of the stress-strain curve (within 1% strain). As a control group, the tensile modulus of the bulk PCL was measured using the abovementioned process. The tensile moduli of PCL and composite (PCL/nHA 10 wt%) bulk were measured as 327.7±2.0 MPa and 398.7±15.6 MPa, respectively. Therefore, according to our result and the reported study [28], the Young's modulus and Poisson's ratio for composite material were assumed to be 398.7 MPa and 0.38, respectively, in the numerical analysis.

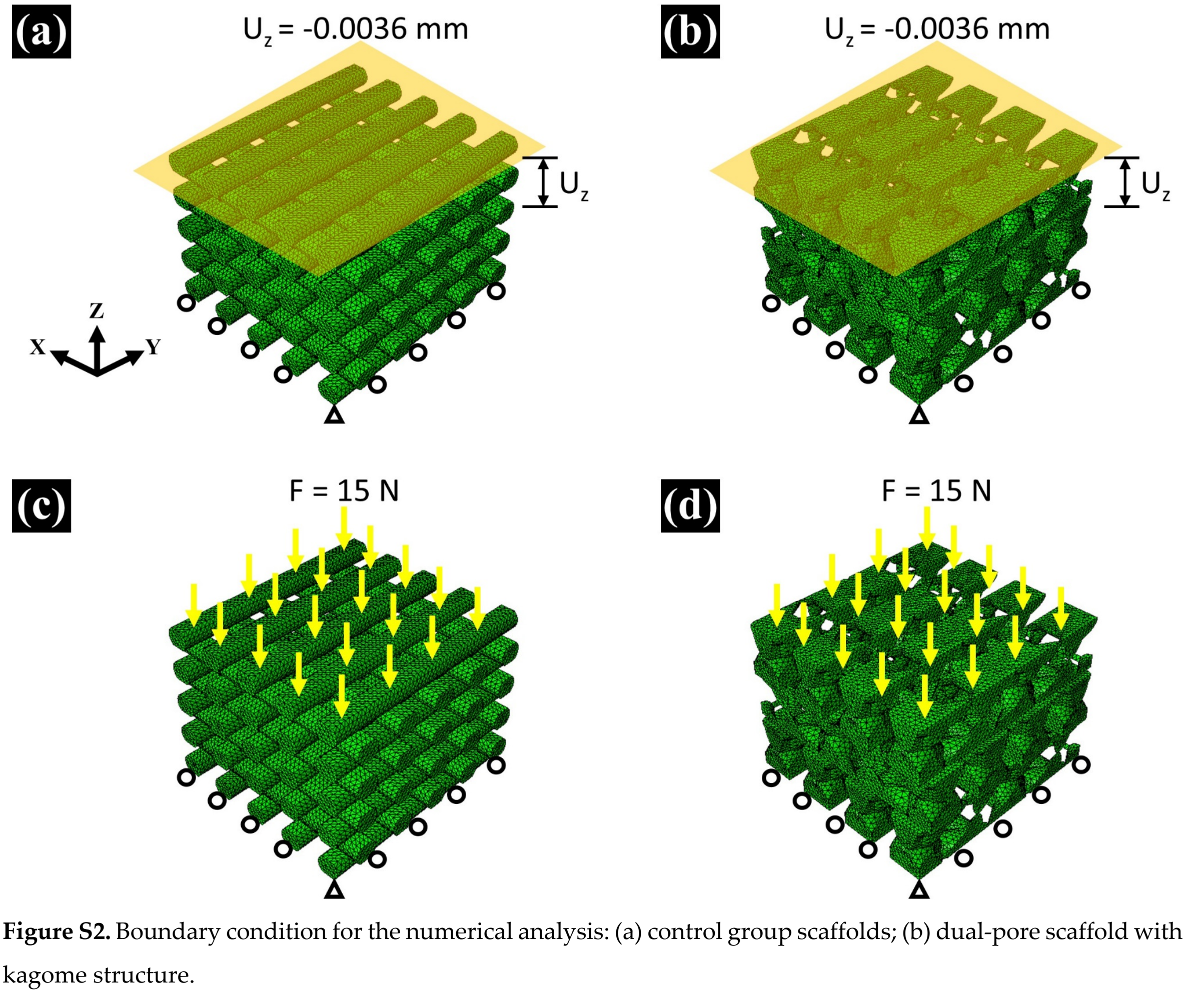

Figure S2. Boundary condition for the numerical analysis: (a) control group scaffolds; (b) dual-pore scaffold with kagome structure.

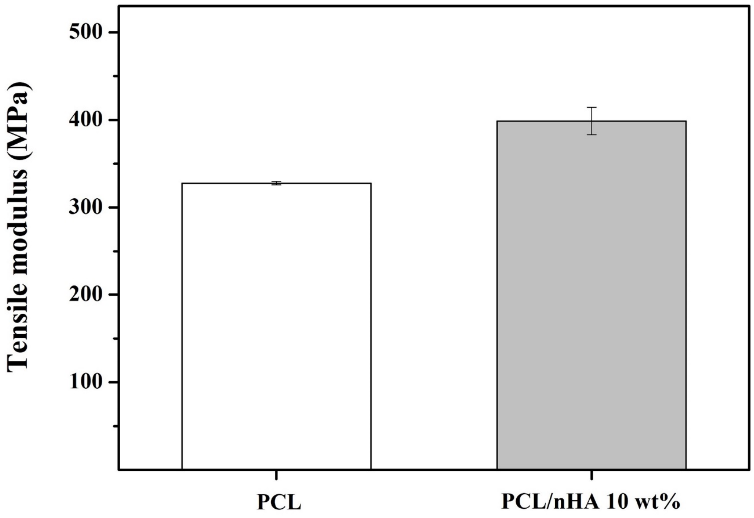

Figure S3. Tensile moduli of bulk PCL and composite bulk (PCL/nHA 10 wt%).

Chapter 2. Numerical analysis of the dual-pore scaffolds with various strand sizes

To investigate the design of the dual-pore scaffold whose compressive modulus is similar to that of the 3D-printed scaffold having a conventional pattern at the same pore size, a numerical analysis of the dual-pore scaffolds with various strand sizes was performed, as depicted in Figure S4. The compressive moduli of dual-pore scaffolds with same pore size was predicted as 26.1 MPa (strand size: 0.8 mm), 34.8 MPa (strand size: 1.0 mm), 58.2 MPa (strand size: 1.2 mm), and 65.4 MPa (strand size: 1.4 mm). The numerical compressive modulus of the dual-pore scaffold with a strand size of 1.4 mm resembled that of the 3D-printed scaffold with the conventional pattern at the same pore size. Therefore, a dual-pore scaffold with 1.4 mm strand size was selected for this study.

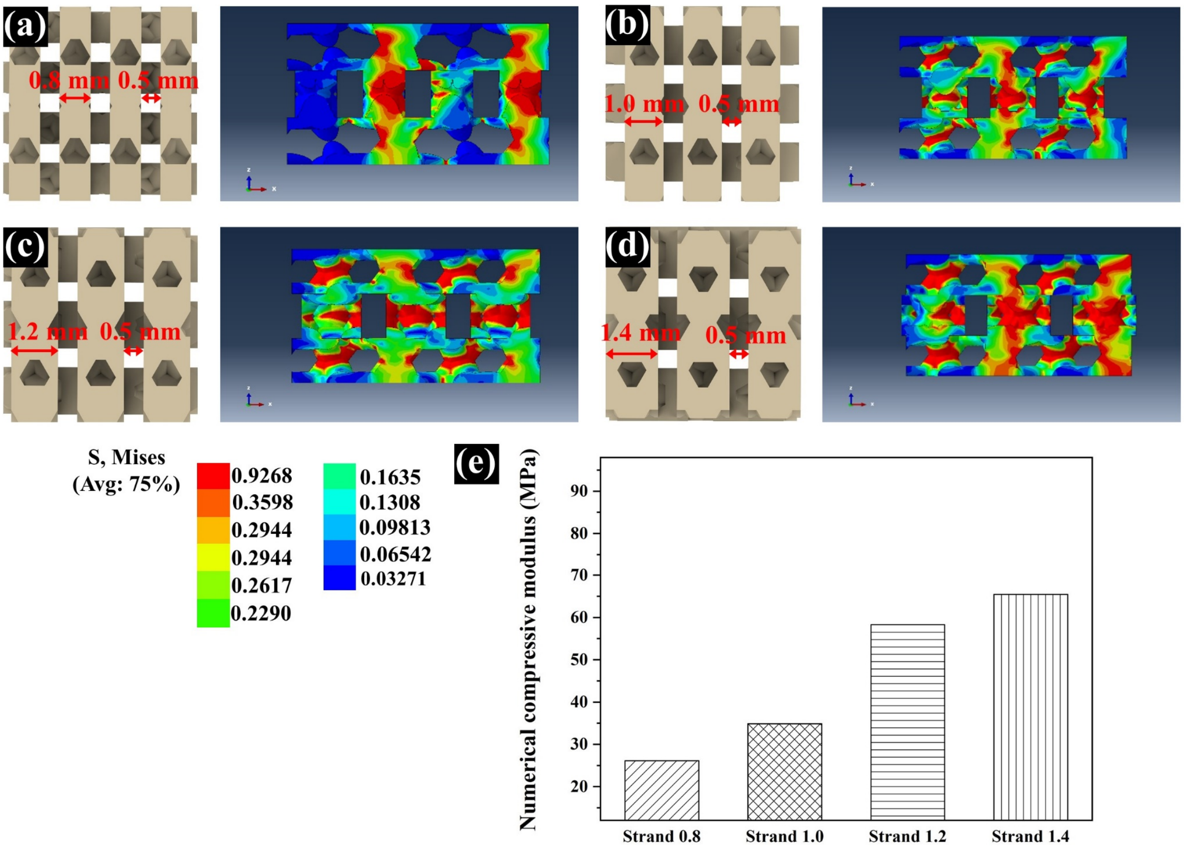

Figure S4. Numerical compressive moduli of dual-pore scaffolds with various strand sizes.
